# Sexual Dimorphism of Pupae and Adults of the Cocoa Pod Borer, *Conopomorpha cramerella*

**DOI:** 10.1673/031.011.5201

**Published:** 2011-04-18

**Authors:** Francisco J. Posada, Ike Virdiana, Maisin Navies, Monica Pava-Ripoll, Prakash Hebbar

**Affiliations:** ^1^ IPM, Tropical Pernnial Crop Consultant, Laurel, MD. USA; ^2^ Sumatra Bioscience. Bah Lias Research Station. Jl Jend A Yani No 2 PO Box 1154. Medan 20111 Indonesia; ^3^ Malaysian Cocoa Board, Tawau, Sabah, Malaysia, Cocoa Research and Development Center, 60237, Tawau, Sabah, Malaysia; ^4^ University of Maryland, Department of Entomology. 4112 Plant Sciences Building, College Park, MD. 20742; ^5^ US Department of Agriculture-APHIS-PPQ, Riverdale, MD, USA

**Keywords:** *Nephelium lappaceum*, south Asia, *Theobroma cacao*

## Abstract

This paper describes the main distinguishing characteristics of female and male pupae and adults of cocoa pod borer, *Conopomorpha cramerella* (Snellen) (Lepidoptera: Gracillariidae). Two pairs of tubercles present on the sterna of segments IX and X of the female pupae are useful in differentiating female from male pupae. The female genital opening is located anterior to the first pair of tubercles and forms a plateau in which the center has a light brown longitudinal depression that indicates the female genital opening. The male genital opening is a conspicuous, brown, longitudinal slit located between the two pairs of tubercles. The sex of the adult moth can be determined by examining the ventrocaudal segments of the abdomen. The last segment of the female abdomen is white, compressed laterally and at the tip, and the hairy anal papillae can be seen. In the male, the ventrocaudal end of the abdomen is black and robust. This information will be useful for laboratory and field diagnosis and while working on sex ratios of this important pest of cocoa.

## Introduction

The cocoa pod borer pest, *Conopomorpha cramerella* (Snellen) (Lepidoptera: Gracillariidae) is of south Asian origin (Bradley 1986). Its primary hosts are plants native to the area such as Rambutam, *Nephelium lappaceum;* Pulasan, *Nephelium mutabile;* Kasai, *Pometia pinnata;* Cola, *Cola nitida, C. acuminata;* and Nam-nam, *Cynometra cauliflora* (Lim et al. 1987).

The eggs laid on the pod surface hatch, and the insect larva bore inside the pods. The larvae feed on the placenta and cause severe damage to the beans. The last instar leaves the pod and pupates outside on damaged pods, leaves, bark or in the soil (Lim et al. 1987).

With the introduction of cocoa *Theobroma cacao* L. (Malvales: Sterculiaceae) to this geographic region, *C. cramerella* moved onto this crop and exploited *T. cacao* as a new host. Since 1986, *C. cramerella* has become the most serious insect pest of cocoa in southeast Asia (Indonesia, Philippines, Malaysia, and New Guinea). Crop losses due to this insect are up to 80% in some geographical regions. By the year 2000, *C. cramerella* was widespread in Indonesia and the yield loss was estimated to be approximately $ 500 million US dollars per year and in Malaysia the production was affected seriously (ICCO 2009).

Understanding the insect biology, behavior, and important morphological characteristics are all important aspects for both basic and applied research in the development of any method of pest control. The ability to distinguish male from female insects is not only important during artificial rearing and developing sterile insect technology, but also while carrying out studies such as pesticide screening, biological control, and changes in sex-ratios and behavior response due to semiochemicals and/or mass trapping.

Little is known about the sexual dimorphism of either pupae or adults of *C. cramerella.* In Gracillaridae, sexual distinction of some species is essential because they have become economically important insect pests (e.g. *Phyllocnistis citrella* and *Cameraria ohridella* (Gracillariidae) [Jacas and Garrido 1996; Freise and Heitland 1999]). The sex in adult Lepidoptera can be determined by color, body shape, antenna shape and length, abdomen size or shape, wing pattern, hair or spurs on the legs. Sometimes it is necessary to sex immature stages such as pupae to separate sexes in order to obtain unmated material to test the response to different treatments such as mating behavior and pheromone studies.

In the family Gracillariidae there is little information on the characteristics that separate female and male pupae and adults. In *P. citrella*, a “row of black scales extending on the seventh abdominal segment” that is present on females but not in males may help indicate the sex (Jacas and Garrido 1996), while in *C. ohridella* the presence of “a rim of chitin” over the distal part of the seventh segment in the male is absent in the female (Freise and Heitland 1999).

The objective of this study is to identify and validate characteristics that allow recognizing *C. cramerella* sexual dimorphism and demonstrate methods that can be used to easily and accurately distinguish male and female specimens in both the pupal and adult stages.

## Materials and Methods

Pupae of *C. cramerella* were collected in Malaysia and Indonesia from several cocoa farms and also from host plants such as *N. lappaceum.* The pupae were collected by carefully excising host tissue (such as leaves, stem, or cocoa pod) on which the cocoon was attached, thereby encompassing the entire cocoon without disturbing it. The individual samples were placed in vials with 70% alcohol along with information about the collection.

The samples were sent to USDA-ARS-Insect Biocontrol Laboratory (IBL) in Beltsville, MD, where the pupal morphology was observed, photographed, and illustrated. The focus was on the most important features such as pupal case length and width, antenna length, posterior leg, and wing position and presence of any special features that might indicate sexual dimorphism.

Additionally, three shipments of live pupae were sent from Malaysia to USDA-ARS-IBL. This material was used to evaluate the morphological characters and to separate the sex of pupae, exuviae, and adults. All pupae were held until adult emergence and some of the adults were subjected to the antennogram for pheromone studies. After the adults emerged, they were checked to confirm that the pupal sex determination matched the adult sex determination by using the last segment configuration method. The adults were also examined to identify any sexual characteristics.

**Figure 1.**
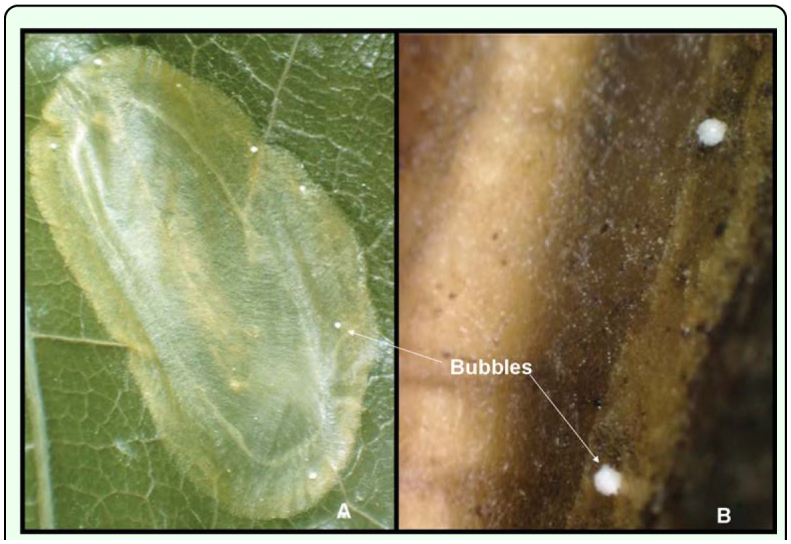
(A) *Conopomorpha cramerella* cocoon with scattered white bubbles. (B) Close-up of white bubbles. High quality figures are available online.

The evaluation of the *C. cramerella* pupal morphological characters to differentiate female from male was conducted by two different individuals. The first evaluation was done at MCB (Malaysian Cocoa Board) Laboratory in Tawau, Malaysia and the second one at USDA-ARS-IBL using the same pupae and morphological characters. When the adults emerged after arrival at the USDA lab, both the exuviae and the adult sex were confirmed to verify whether the criteria and the methods used to separate the sexes were accurate.

## Results and Discussion

### *Conopomorpha cramerella* pupae sexual dimorphism

*C. cramerella* pupae were collected in the field from leaves, pods, bark, soil crevices, and leaf litter. *C. cramerella* larvae pupate inside a cocoon that is built with transparent fine brown silk. The cocoon is constructed with an initial layer of silk attached to the surface over which the cocoon is placed and to this is attached another layer that covers the pupa. The cocoon is oval in shape, smooth, and brown in color with a darker, more thickened margin. The pupa occupies about three quarters of the entire cocoon (Figure 1A).

Several small, white bubble-like spheres about (Mean ± SE) 0.3 ± 0.0 mm in diameter on average (n = 20) are attached to the top surface of the cocoon. Three to eight bubbles are scattered and distributed over the edges of the cocoon (Figure 1B). The function of these bubbles is unknown. There is speculation that they are ornamental and that may protect the pupae from natural enemies by functioning as objects of distraction (Wagner et al. 2000).

**Figure 2.**
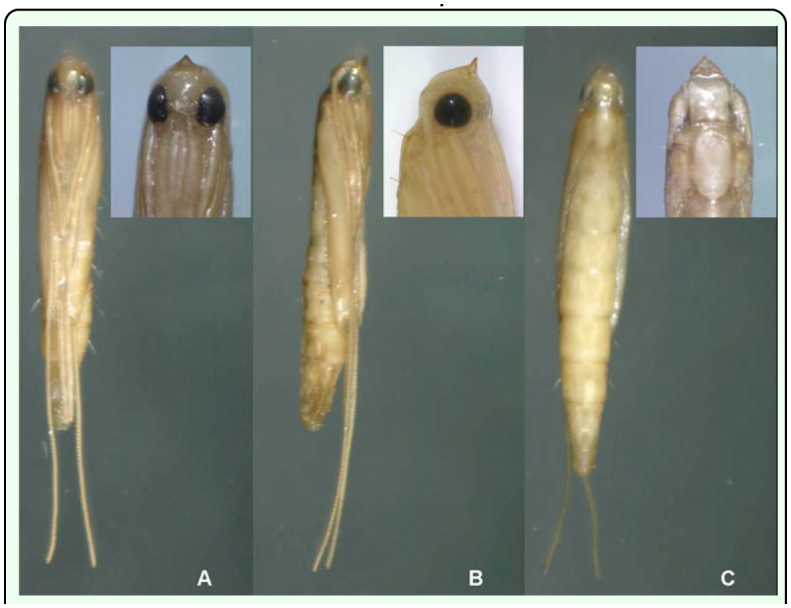
*Conopomorpha cramerella* pupae with a magnification of the beak. (A) Ventral view. (B) Lateral view. (C) Dorsal view. High quality figures are available online.

**Figure 3.**
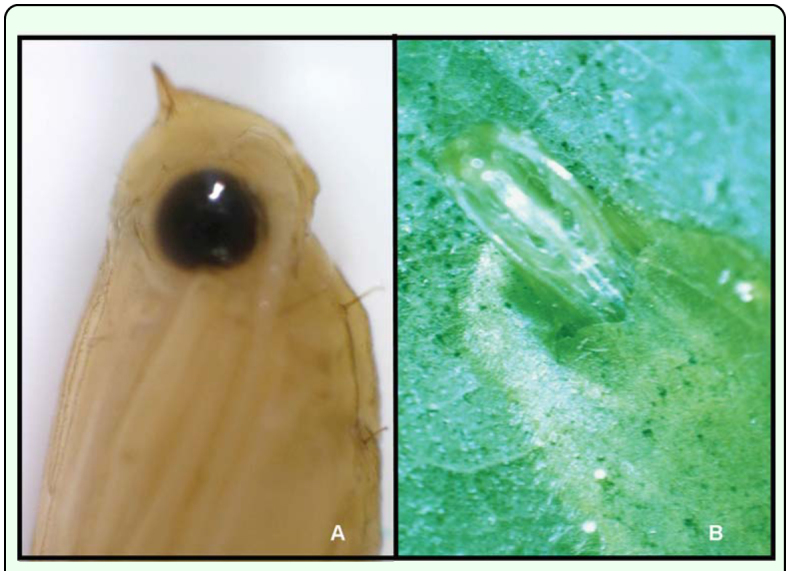
(A) Pupae head of *Conopomorpha cramerella* showing the peak cutter. (B) Exuviae hold on the open cocoon after adult emergence. High quality figures are available online.

The pupa is yellowish, slender with prominent eyes that turn black when the pupa is in the final stage of development (Figure 2A–C). The pupal length, including the body and the antenna, is 7.2 ± 0.5 mm on average (n = 59). The pupal length (body) is 4.6 ± 0.6 mm on average (n = 59) and the antennae extend 2.6 ± 0.4 mm on average from the rear tip of the pupa (n = 59).

The pupa and its appendices vary in length and width. This variation was thought to be useful as a means to separate the sexes, but their allometry, in this study, did not indicate a reliable means to distinguish males from females.

The pupal head possesses a prominent frontal process that arises from the front. It is ornamented with ridges and pointed in the middle. Viewed laterally, the frontal process makes an angle under 30°. The process is directed dorsally and has a triangular shape (Figure 2A–C, 3A).

When the pupal stage is completed this process is used to cut open the cocoon allowing the adult to eclose from the pupal case, which remains half inside the cocoon (Patocka and Zach 1995; De Prins et al. 2003) (Figure 3A, B).

**Figure 4.**
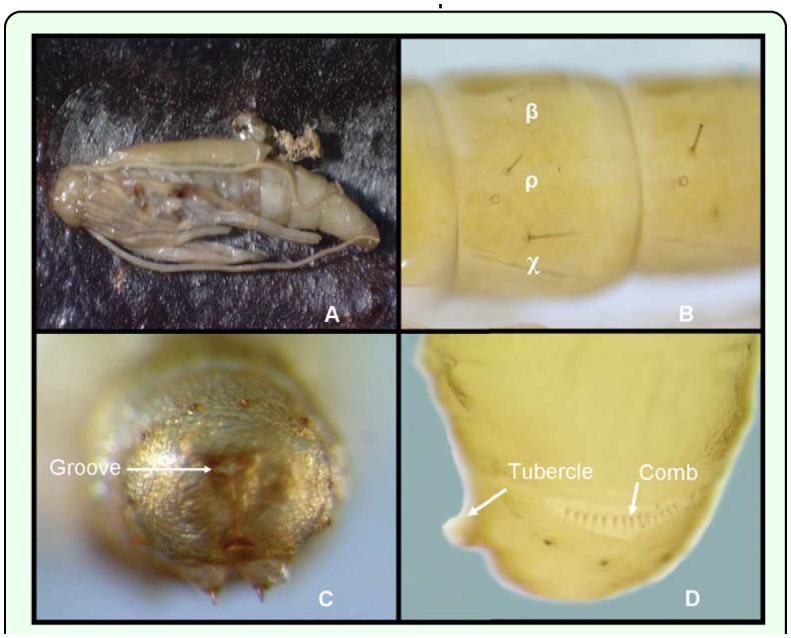
(A) A *Conopomorpha cramerella* pupa with its append spread out. (B) *C. cramerella* pupal spiracle. (C) Caudal view showing the crown of ten tubercles and the anal groove. (D) Lateral view showing a comb on the posterior part of segment nine. High quality figures are available online.

All of the appendices on the pupa - maxillary palpi, labial palpi, proboscis, legs, wings, and antenna - can be seen clearly. The antenna and posterior legs and wings are free and not attached to the pupal case (Mosher 1969). This feature makes it resemble an exarate pupal type more than an obtect pupal type (Figure 4A). The antenna is longer than the caudal apex of the abdomen and is nearly 1/3 X longer than the entire pupa; it also is longer than the apices of the posterior legs.

The maxillary palpi, labial palpi, proboscis, and fore and middle legs appear attached to one another, but are actually free and not fused to the pupal case as typical in obtect pupae (Mosher 1969).

The maxillary palps, labial palps, and proboscis of the mouth parts are relatively large. The apex of the proboscis is longer than the forelegs. The wings reach the 4th or 5th abdominal segments.

The prothorax possesses two spines placed laterally and close to the head. Similar, caudally directed spines are present on the meso and metathorax.

The pupa has six visible pairs of circular spiracles possessing a dark brown peritremme common to many microlepidoptera (Peterson 1979). On each abdominal segment there are a group of setae located posterior to the spiracle. Figure 4B shows the first setae β located on the subdorsal area, the second setae ρ located on the supraspiricular area, and the third setae X, the largest of the three, located on the subspiracular area (Figure 4B).

The caudal segment has a ring, or crown, consisting often small tubercles without setae. Two prominent brown tubercles end in a tooth oriented forward. This pair of tubercles, with it is recurved spines, may represent the cremaster (Davis and Robinson 1999) even though it is not present on the pupal apice as typically occurs in other Lepidoptera. The abdominal apex is round and with a ventral anal groove (Figure 4C).

On the posterior edge of the ninth segment on the lateral side, there is one structure that resembles a comb that has crochets that are uniserial, arise from one line, and are different lengths (Peterson 1979). This structure in combination with the two prominent tubercles on the ventral part may function as a spur to help the adult escape from the pupal case during emergence. Additionally, these structures can be used as a reference landmark to locate the sexual defining characteristic on the ventral site in females and males (Figure 4D).

Sexual dimorphism is difficult to establish in *C. cramerella* using the current morphological characteristics that are presented and used to distinguish sex in Lepidoptera including the Geometridae, Noctuidae, Pyralidae, or Saturnidae.

**Figure 5.**
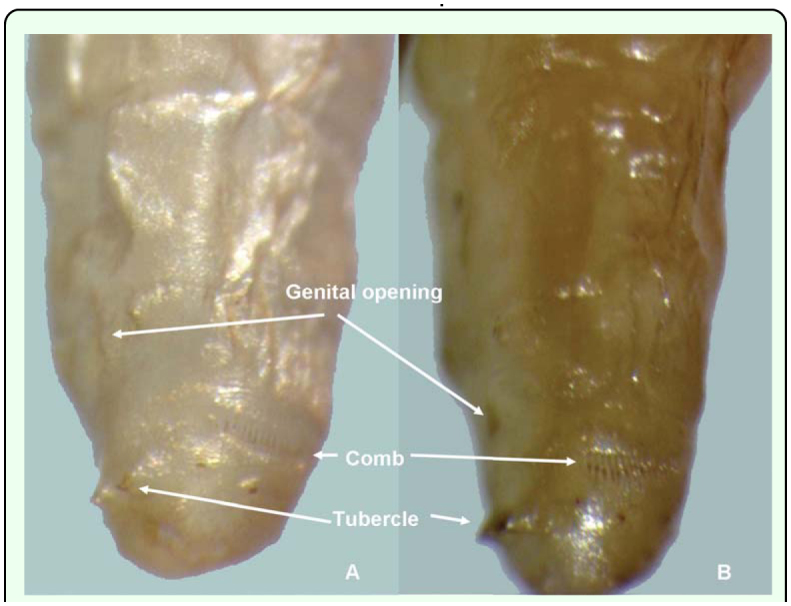
Lateral view of segments **VII** – X of *Conopomorpha cramerella* pupae showing the structures to locate the genital opening. (A) Female. (B) Male. High quality figures are available online.

**Figure 6.**
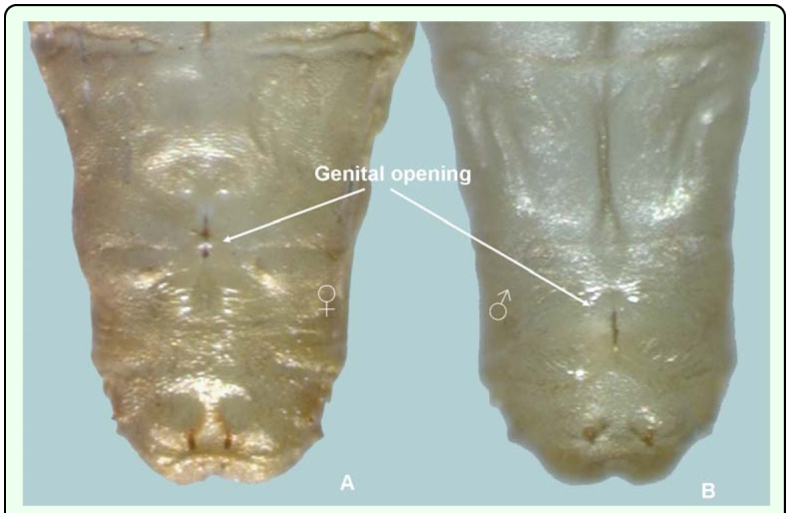
Sexual dimorphism of *Conopomorpha cramerella* pupae. (A) Female. (B) Male. High quality figures are available online.

The sexual characteristics used to differentiate the dimorphism between female and male pupae of *C. cramerella* are visible when viewed from the ventrum (Figures 5, 6). The female genital opening or slit is located between the eighth and ninth segment and above the two marginal tubercles located on segment nine. In the eighth segment, a plateau can be seen and on the seventh segment, a longitudinal ridge is present that is also present on anterior segments (Figures 5A, 6A). The male genital opening is located in the middle of segment nine and can be found between the two marginal tubercles present on the anterior part of segment nine, and the two tubercles present on segment ten are located together on the ventrum with prominent spines (Figures 5B, 6B).

### Sexual dimorphism of *Conopomorpha cramerella* adults

**Figure 7.**
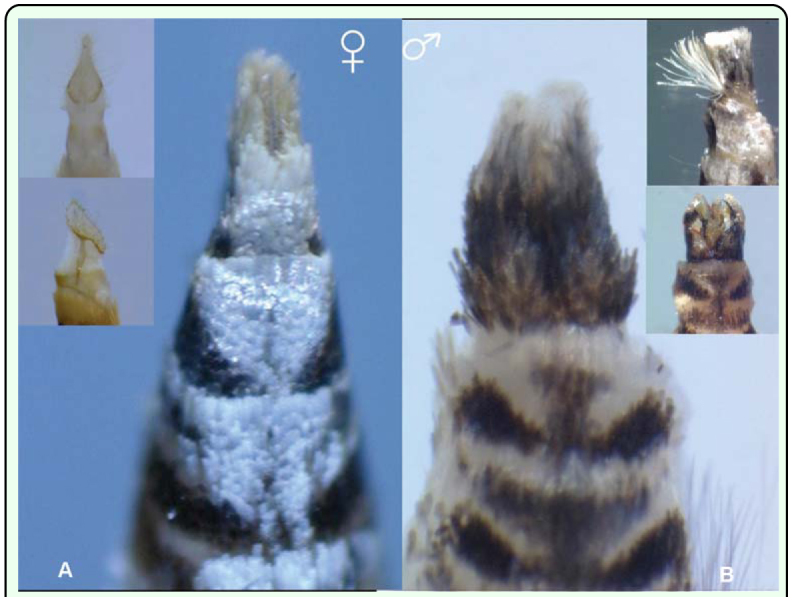
Ventral view of sexual dimorphism of *Conopomorpha cramerella* adult. (A) Female with the ovipositor showing the anal papillae and (B) male showing the vulvae and the hair pencil. High quality figures are available online.

The adult female and male are similar in possessing the same color and wing pattern. However, it is not necessary to remove the scales in order to determine sex. The caudal apex female abdomen is characteristic in being compressed laterally, with white sterna. The hairy anal papilla of the ovipositor can be seen (Figure 7A). The caudal segment of the male abdomen is black and broader. Also, it is possible to observe the valva of the male genitalia (Figure 7B). For further information on *C. cramerella* genitalia see Bradley (1986).

Immediately following emergence, females were observed to be very active while in a box with cocoa leaves with males nearby enclosed in vials. Females walked toward and rubbed the surface of the leaves with the tip of the abdomen. This is probably a behavior to release the pheromone and call for males. This behavior can also be another means to differentiate females from males.

### Evaluation of morphological characters to separate pupae and adult by sex.

The sexual characters used to separate C. *cramerella* females and males described above were evaluated on 164 live pupal samples received in a shipment from Malaysia for the purpose of conducting antennogram pheromone studies. Sex determination using abdominal structures discussed above was 95% consistent in separating the pupae and 100% for exuviae and adults. These results showed that *C. cramerella* sexual dimorphism can be used to separate pupae, exuviae, and adults.

In order to achieve high precision in the ability to sex *C. cramerella* it is necessary that people involved in this activity have the proper training and good visual references. By using the morphological characteristics of *C. cramerella* described in this paper, it will be possible, when collecting pupae or exuviae from the field, to estimate its population dynamics, to determine sex radios and to correlate effects of male capture with pheromone traps and its anticipated effect on the population. This information should assist in developing and evaluating new alternative methods of research on *C. cramerella.*
